# Protein Kinase C: Targets to Regenerate Brain Injuries?

**DOI:** 10.3389/fcell.2019.00039

**Published:** 2019-03-20

**Authors:** Noelia Geribaldi-Doldán, Ricardo Gómez-Oliva, Samuel Domínguez-García, Pedro Nunez-Abades, Carmen Castro

**Affiliations:** ^1^Área de Fisiología, Facultad de Medicina, Universidad de Cádiz, Cádiz, Spain; ^2^Instituto de Investigación e Innovación Biomedica de Cádiz (INIBICA), Cádiz, Spain; ^3^Departamento de Fisiología, Facultad de Farmacia, Universidad de Sevilla, Seville, Spain

**Keywords:** protein kinase C, neurogenesis, ADAM17/TACE, brain injury, neuroregeneration

## Abstract

Acute or chronic injury to the central nervous system (CNS), causes neuronal death and irreversible cognitive deficits or sensory-motor alteration. Despite the capacity of the adult CNS to generate new neurons from neural stem cells (NSC), neuronal replacement following an injury is a restricted process, which does not naturally result in functional regeneration. Therefore, potentiating endogenous neurogenesis is one of the strategies that are currently being under study to regenerate damaged brain tissue. The insignificant neurogenesis that occurs in CNS injuries is a consequence of the gliogenic/non-neurogenic environment that inflammatory signaling molecules create within the injured area. The modification of the extracellular signals to generate a neurogenic environment would facilitate neuronal replacement. However, in order to generate this environment, it is necessary to unearth which molecules promote or impair neurogenesis to introduce the first and/or eliminate the latter. Specific isozymes of the protein kinase C (PKC) family differentially contribute to generate a gliogenic or neurogenic environment in injuries by regulating the ADAM17 mediated release of growth factor receptor ligands. Recent reports describe several non-tumorigenic diterpenes isolated from plants of the *Euphorbia* genus, which specifically modulate the activity of PKC isozymes promoting neurogenesis. Diterpenes with 12-deoxyphorbol or lathyrane skeleton, increase NPC proliferation in neurogenic niches in the adult mouse brain in a PKCβ dependent manner exerting their effects on transit amplifying cells, whereas PKC inhibition in injuries promotes neurogenesis. Thus, compounds that balance PKC activity in injuries might be of use in the development of new drugs and therapeutic strategies to regenerate brain injuries.

## Neurogenesis in the Adult Brain Under Physiological Conditions

Cell replacement in several mammalian organs is an orchestrated process that may lead to the regeneration of a completely functional organ. Unfortunately, this is not the case of the adult central nervous system (CNS). Neurogenesis, the process of generation of new neurons that occurs during development of the CNS and remains during the infant and adult stages is built up on the capacity of neural stem cells (NSC) to produce neurons and glial cells. Yet quiescent NSC are distributed ubiquitously along the adult CNS (Magavi et al., 2000), once development is ended, neurogenesis is restricted to a few specific regions. In these areas NSC produce neurons because they are situated in a context of signaling molecules that induce their transition to an activated state, from which they produce a progeny mainly comprised of cells with a neuronal phenotype. Two neurogenic regions have been thoroughly described in the adult mammalian brain, the subventricular zone (SVZ) and the dentate gyrus of hippocampus (DG) (Kuhn et al., 1996; Doetsch et al., 1997). Within these regions an environment of extracellular signaling molecules creates a neurogenic niche that preserves the necessary conditions to support neurogenesis during a lifetime. Different cell types derived from the NSC progeny can be distinguished within these niches: undifferentiated neural progenitor cells (NPC) produced by activated NSC, and neuronal progenitor cells (neuroblasts) that differentiate into mature neurons. Since the potentiality of NPC is almost identical to that of NSC, they can produce either neuronal progenitors or glial progenitors (Reynolds and Weiss, 1992; Doetsch et al., 1999; Torroglosa et al., 2007). However, once in the niche, extracellular, matrix-bound and membrane-bound signals determine their fate toward a neuronal phenotype (Codega et al., 2014).

## Injury-Induced Neurogenesis

Adult neurogenesis has generated a great deal of attention in the context of designing cell replacement therapies following neuronal loss. Focal traumatic or cerebrovascular brain injuries cause acute damage, induce neuronal death and irreversible cognitive deficits or sensory-motor alterations (Blennow et al., 2012). No effective treatment is currently available to compensate neuronal loss in these patients, however, the potential of the CNS to generate new neurons that replace the lost ones has opened a new perspective in the development of therapies to treat this type of lesions. One of the strategies, currently under study, to enable neuronal replacement is facilitating the recruitment of endogenous NPC and neuroblasts within the injured tissue (Saha et al., 2012). Two different sources of neuroblasts facilitate neuronal replacement in injuries: NPC generated from NSC activated at the site of injury (Magavi et al., 2000; Chen et al., 2004; Geribaldi-Doldan et al., 2018) and NPC or actual neuroblasts that are generated in neurogenic regions as a reaction to the damage, which migrate in the direction of the injury (Parent et al., 1997; Arvidsson et al., 2002; Kandasamy et al., 2015; Geribaldi-Doldan et al., 2018). However, cells from both sources would need an adequate environment to survive and to lead their destiny toward mature neurons that would integrate into existing neuronal circuits (Romero-Grimaldi et al., 2011). Following an injury, neurogenic regions react activating different steps of the neurogenic process: activation of quiescent NSC, induction of NPC proliferation, differentiation, and alteration of neuroblast migration patterns (Liu et al., 1998; Fallon et al., 2000; Jin et al., 2001) in the direction of the injured area. However, this attempt to repair the damaged tissue is generally unsuccessful and most of the originated NPC lead the destiny of their progeny toward astroglial cells (gliogenesis) rather than neurons (neurogenesis) (Romero-Grimaldi et al., 2011; Susarla et al., 2014; Geribaldi-Doldan et al., 2018). The lack of significant neurogenesis in damaged brain areas, even when endogenous NPC are available, may be due to the absence of molecules necessary for neuronal differentiation and/or to the presence of molecules that favor the differentiation of NPC toward a glial phenotype (Benner et al., 2013). Inflammatory molecules released mainly by glial and microglial cells create a gliogenic/non-neurogenic environment that facilitates the generation of glial progenitor cells, which differentiate into glial cells (Buffo et al., 2008). Thus, it may be possible that these glial cells activate a paracrine positive feedback loop that favors gliogenesis from NPC over neurogenesis.

## Neuronal Replacement Strategies: Promotion of Endogenous Neurogenesis

Potentiating endogenous neurogenesis is one of the strategies that are currently being under study to regenerate damaged brain tissue. Four important stages of the neurogenic process can be modulated in order to promote endogenous neurogenesis: (1) to stimulate NSC activation and proliferation of NPC within the lesion (Luzzati et al., 2014; Barker et al., 2018), (2) to generate an environment that favors neurogenesis in neurogenic regions to lead the fate of NPC into a neuronal phenotype (Parent et al., 1997; Grade and Gotz, 2017), (3) to promote migration of neuroblasts toward the damaged areas, as well as survival (Geribaldi-Doldan et al., 2018), and (4) to stimulate differentiation of neuroblasts into mature neurons (Geribaldi-Doldan et al., 2018) facilitating the posterior integration of the newly generated neurons into preexisting circuits. In summary, replacement of dead neurons in an injured CNS region requires the modification of the extracellular environment to generate a neurogenic niche in which the progeny of endogenous NSC is predominantly mature functional neurons. However, it is first necessary to uncertain which molecules promote or impair neurogenesis and introduce the first and/or eliminate the latter. Recent reports aimed to understand the cellular and molecular mechanisms involved in the generation of this gliogenic/non-neurogenic background highlight the key role of neurogenic signaling molecules such as Noggin (Lim et al., 2000) or Neuregulin 1 (NRG1) that promotes neuroblast migration, and gliogenic signaling pathways such as those initiated by Notch (Benner et al., 2013), or the epidermal growth factor receptor (EGFR) (Kuhn et al., 1997; Gonzalez-Perez et al., 2009; Romero-Grimaldi et al., 2011; Geribaldi-Doldan et al., 2018) among other signals.

It is worth mentioning the essential role of EGFR activation in the neurogenic processes. EGFR signaling participates in SVZ neurogenesis promoting proliferation of undifferentiated transit amplifying progenitors (TAPs) (Doetsch et al., 2002; Torroglosa et al., 2007). In NPC cultures under differentiation conditions, EGFR activation favors gliogenesis over neurogenesis (Romero-Grimaldi et al., 2011). In brain injuries, the shedding of EGFR ligands such as TGFα activate EGFR and prevent the generation of neurons facilitating gliogenesis and contributing to the generation of gliogenic niches in areas of brain damage (Romero-Grimaldi et al., 2011; Geribaldi-Doldan et al., 2018). Regulating EGFR activity might be relevant when developing strategies to promote endogenous neurogenesis in brain injuries and understanding the mechanisms that activate this receptor may lead to the identification of molecular targets to regenerate brain injuries. Thus, in the subsequent paragraphs, we will discuss molecular mechanisms involved in the regulation of EGFR activity.

## ADAM17/TACE Dependent EGFR Ligand Release

**FIGURE 1:**
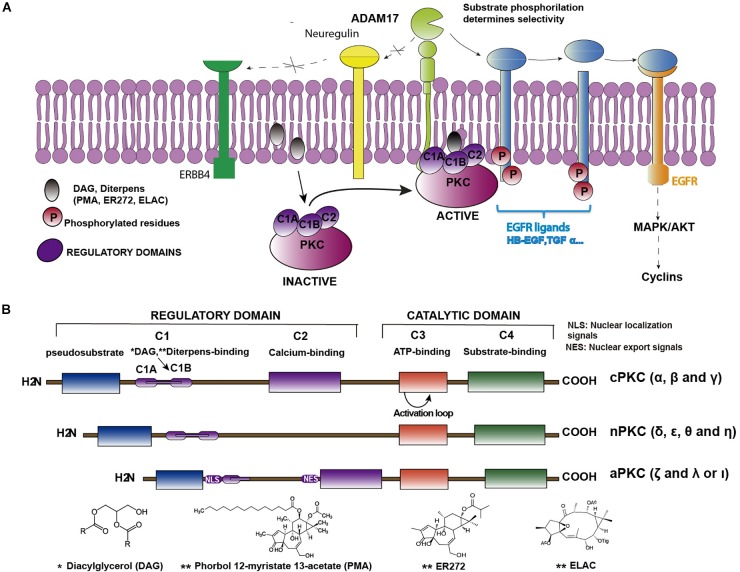
Structure of PKC isozymes and their indirect role in EGFR activation. **(A)** Cartoon representing the sequence of PKC-ADAM17-TGFα-EGFR pathway. Binding of DAG or non-physiological diterpenes to the regulatory domains of PKC activates the enzymes. Upon activation, enzymes are translocated close to the plasma membrane where they catalyze the phosphorylation of membrane bound EGFR pro-ligands or ligands of other receptors of the ERBB family (i.e., neuregulin) or ligands that activate other receptors, (i.e., neuregulin, which activates ERBB4). Only phosphorylated pro-ligands are selected by ADAM17 as substrates over other non-phosphorylated ones. ADAM17-mediated shedding occurs on the phosphorylated pro-ligands, releasing the soluble ligand and activating the receptor. **(B)** Classification of PKC isozymes according to their structure and regulatory properties. Regulatory domains (C1 and C2) and binding sites for regulatory molecules (DAG, Ca^2+^, and PS) are shown as well as the conserved catalytic domains (C3 and C4). ^∗^See structure of Diacylglicerol below; ^∗∗^See structures of different diterpenes (PMA, ER272 and ELAC) below.

As commented above, epidermal growth factor (EGF)–induced EGFR activation, in cultures of NPC isolated from the SVZ, promotes glial cell differentiation whereas EGFR inhibition facilitates the differentiation of these cells toward a neuronal phenotype (Romero-Grimaldi et al., 2011). The effect of the EGFR inhibition can be mimicked *in vitro* by the inhibition of the tumor necrosis factor alpha-converting enzyme (ADAM17/TACE), a metalloprotease of the A Disintegrin And Metalloproteinase (ADAM) family. These metalloproteases catalyze the cleavage of the extracellular domains (ectodomains) of several EGFR ligands. These ligands are synthesized as membrane-anchored precursor proteins (pro-ligands). The active soluble ligands are detached from the pro-ligands and released to the extracellular medium in a proteolytic reaction catalyzed by convertases of the ADAM family. In particular TGFα release is mediated by ADAM17 (Blobel, 2005), a membrane-bound peptidase, which is a limiting step in regulating signaling through EGFR. ADAM17 is the main convertase involved in the release of the EGFR ligands TGFα, HB-EGF, Epiregulin and Amphiregulin (Sunnarborg et al., 2002; Lee et al., 2003). Nevertheless, ADAM17 also catalyzes the release of other ligands of the ErbB family of receptors (Figure 1A). The selectivity of this enzyme for each ligand is mediated by phosphorylation reactions in the C-terminal domain of the pro-ligand, catalyzed by kinases of the protein kinase C (PKC) family (Dang et al., 2013).

## PKC Isozymes Structure and Classification

Kinases of the PKC family are enzymes composed of regulatory and catalytic domains (Figure 1B), which phosphorylate a great variety of substrates. These proteins remain in an inactivated state that can be reverted upon the binding of their regulators diacylglycerol (DAG), calcium and phosphatidyl serine (PS) to the regulatory domains. They are characterized by a conserved kinase domain, which undergoes a conformational change and activates itself to enable catalysis. As shown in Table 1, kinases of the PKC family play an essential role in transducing signals related with cell cycle entrance, differentiation, apoptosis or autophagy among other functions (Watanabe et al., 1992; Dempsey et al., 2000; Black and Black, 2012). This family of proteins consists of ten serine-threonine kinases, which based on their regulatory domains and physiological activators, are classified in three subfamilies (Mellor and Parker, 1998): the classical, the novel and the atypical. The classical PKCs (α, β, and γ), depend on calcium, DAG, and PS for their activation. The novel PKCs (δ, ε, θ, and η), are calcium independent kinases but they still require DAG and PS for their activation. The atypical PKC (λ and ζ) do not depend on calcium or DAG for their activation and are regulated by protein-protein interactions (Rosse et al., 2010; Figure 1B).

**Table 1:** Pathophysiological role of PKC isozymes.

PKC isoforms	Tissue expression	Functions	Reference
**Classical PKCs**			
α	Ubiquitous	• Cell proliferation and metastasis Heart failure, decreased contractility. • Apoptosis, tumorigenecity, cell adhesion, differentiation, migration. • Synaptic defects in AD. • Role in learning and memory.	Konopatskaya and Poole, 2010; Liu and Molkentin, 2011; Sun and Alkon, 2014; Alfonso et al., 2016; Singh et al., 2017
β	Ubiquitous	• Cancer development: vasculogenesis and cell invasion. • Diabetes: vascular complications.	Sledge and Gokmen-Polar, 2006; Geraldes and King, 2010
γ	Brain and spinal cord	• Pain modulation in dorsal root ganglia. • Long term potentiation, long term depression, modulation of receptors, neurological disorder.	Saito and Shirai, 2002; Sweitzer et al., 2004
**Novel PKCs**			
δ	Ubiquitous	• Proliferation, immune function, apoptosis, and cell migration. • Fertility. • Cancer development: angiogenesis. • Regulation of amyloid-β degradation pathway. • Neuronal loss in animal models of Parkinson’s Disease	Zhang et al., 2007; Reyland, 2009; Basu and Pal, 2010; Ma et al., 2015; Du et al., 2018
ε	Ubiquitous	• Heart failure, increased fibrosis, ischemia, mitochondria protection. • Pain modulation in spinal cord. • Bipolar diseases: neuronal transmission malfunction. • Role in learning and memory. • Ischemic tolerance	Sweitzer et al., 2004; Inagaki et al., 2006; Einat et al., 2007; Liu et al., 2012; Sun and Alkon, 2014
η	Ubiquitous	• Acquired resistance to radiation. • Epithelial cell growth and differentiation.	Kraft et al., 1982; Cabodi et al., 2000
θ	Ubiquitous	• Gastrointestinal stromal tumor, cell proliferation and antiapoptosis. • T cell responses, inflammation.	Ou et al., 2008; Zanin-Zhorov et al., 2011
**Atypical PKCs**			
λ	Ubiquitous	• Glioblastoma cell invasion.	Baldwin et al., 2008
ζ	Ubiquitous	• Breast cancer cell metastasis. • Glioma, cell proliferation, survival, invasion, and migration.	Van Kolen and Slegers, 2006; Guo et al., 2009; Huang et al., 2012


## PKC and Therapeutic Considerations

The above-mentioned role of PKC isozymes in regulating ADAM17 activity may lead to hypothesize that modulating PKC activity might be of use as a treatment to promote endogenous neurogenesis in lesions. However, therapeutic considerations may be taken into account because of the strong association of these kinases in cancer or neurodegenerative diseases such as Alzheimer’s disease (AD) (reviewed in Newton, 2018a; Table 1). Almost all PKC isozymes have been associated with tumor progression and also with the metastasis process. Classical PKC are involved in tumorigenicity, for example PKCα regulates cell motility in some cancer models and some authors described a relationship between up or down-regulation of these isozyme depending of the type of cancer (Konopatskaya and Poole, 2010). PKCβ II induces endothelial cells proliferation and stimulates tumor angiogenesis in breast cancer, whereby some inhibitors of this isozyme have been postulated as a therapeutic treatment (Sledge and Gokmen-Polar, 2006) for this type of cancer. Novel PKCδ has been associated with pro-apoptotic signaling, in fact it is involved in tumor suppression inhibiting cell cycle progression (Basu and Pal, 2010). PKCε is one of the most studied isozymes in cancer research with a significant role in lung cancer (Baxter et al., 1992). PKCθ has been implicated is gastrointestinal cancer (Ou et al., 2008). Atypical PKC as PKCζ is involved in breast cancer development and in promoting glioma initiating cells proliferation, invasion and migration (Huang et al., 2012; Malla et al., 2012). It is important to clarify that in general, a reduced PKC activity and protein expression has been associated to different types of cancer.

This reduced activity in tumors contrasts with the enhanced PKC activity and expression found in models of neurodegenerative diseases such as AD or stroke (Table 1) (reviewed in Sun and Alkon (2014), Newton (2018a)). Acute and chronic changes in PKC activity can be found in models of AD, stroke and age-dependent neurodegeneration with different effects depending on the disease and the stage of the disease. As the body ages, activity and proper translocation of PKC isozymes is critical for memory, and injury repair (Table 1) (reviewed in Lucke-Wold et al., 2015). Gain of function mutations of classical PKCα activity has been specifically involved in the reduction of synaptic activity caused by AD (Alfonso et al., 2016) contributing to cognitive decline. In a similar fashion, inhibition of novel PKCδ reduces amyloid ß and reverses AD (Du et al., 2018). Alteration on PKC expression can also be observed as aging occurs and a downregulation in PKC expression is found in different models probably as a consequence of epigenetic modifications (Pascale et al., 2007). Whether these alterations are the basis of the disease or a homeostatic response to the disorders remains to be clarified.

## PKC, ADAM17 and EGFR: Role in Adult Neurogenesis

Protein kinase C isozymes phosphorylate several downstream substrates including EGFR ligands as well as other ErbB receptor ligands such as NRG1. Specific activation of PKC isozymes determines ADAM17 selectivity for its different substrates (Figure 1A). Thus, PKCα activated by Phorbol-12-myristate-13-acetate (PMA) catalyzes the phosphorylation of TGFα, Amphiregulin and HB-EGF precursors facilitating their shedding mediated by ADAM17 and releasing the soluble growth factor outside the cell (Dang et al., 2013). On the contrary, activation of novel PKCδ is required for ADAM17 mediated secretion of NRG1. Phosphorylation of serine 286 in the cytoplasmic domain of NRG1 catalyzed by PKCδ facilitates the scission of its ectodomain (Watanabe et al., 1992; Dang et al., 2011) releasing NRG1 into the extracellular medium. Overall, ADAM17 substrate specificity and selectivity is mediated by the activation of different PKC isozymes, which play a key role in the secretion of different types of ligands (Dang et al., 2011, 2013) governing several steps of adult neurogenesis. As examples, autocrine secretion of TGFα in brain injuries leads NPC toward a glial fate preventing the generation of neurons; on the contrary, inhibition of ADAM17 dramatically increases the generation of neurons (Romero-Grimaldi et al., 2011; Geribaldi-Doldan et al., 2018). NRG1 mediated activation of ErbB4 promotes neurogenesis in the adult SVZ increasing NPC proliferation and organizing migration of neuroblasts from the SVZ toward the olfactory bulb (Anton et al., 2004; Ghashghaei et al., 2006). These evidences point out at the mission of specific kinases of PKC family on stimulating the production of signaling molecules such as TGFα or NRG1 (Dang et al., 2011, 2013), which may have a decisive role in leading NSC and NPC toward gliogenesis or neurogenesis, respectively (Ghashghaei et al., 2006; Romero-Grimaldi et al., 2011).

## PKC Isozymes in Adult Neurogenesis

Several members of the PKC family are present in neurogenic regions (Minami et al., 2000) and participate in distinct signaling cascades initiated by growth factors (GF), often determining GF specificity (Corbit et al., 2000). Activation of classical PKCß promotes proliferation of NPC *in vitro* and induces the expression of cyclins E and D in the absence of EGFR. *In vivo* in the SVZ and DG of mice, PKC activation promotes proliferation (Geribaldi-Doldan et al., 2016; Murillo-Carretero et al., 2017) mainly of EGFR^+^ transit amplifying cells. In addition, atypical PKC have been involved in the NSC-to-neuron transition during development, and in the adult brain (Wang et al., 2012). On the contrary, novel PKCε, activation is crucial for the astrocytic differentiation of NPC (Steinhart et al., 2007). Phosphorylation of the CREB binding protein (CBP) by atypical PKC promotes hippocampal neurogenesis as well as memory and learning in mature adult mice in which CREB activity is reduced as a consequence of age (Gouveia et al., 2016). Metformin-induced activation of atypical PKC in mice promotes hippocampal neurogenesis and enhances spatial reversal learning in the Morris Water Maze task (Wang et al., 2012). Furthermore, local treatment of mechanical brain injuries with a pan-PKC inhibitor, promotes neuroblast enrichment facilitating differentiation of NPC toward neurons (García-Bernal et al., 2018). Overall, activation of classical PKC isozymes promotes neurogenesis in neurogenic regions whereas its inhibition facilitates neurogenesis in injuries.

## PKC Activation by Diterpenes

The physiological activator of PKC is DAG; this molecule binds to the C1B domain of classical and novel PKC isozymes (Figure 1B) inducing a conformational change that results in the activation of the protein. The affinity of PKC isozymes for DAG is higher in novel PKC than in classical PKC isozymes (reviewed in Newton, 2018b). Non-physiological molecules such as phorbol esters can activate classical and novel PKC isozymes. These tetracyclic diterpenoids activate PKC because they mimic the action of diacylglycerol (DAG) (Newton, 1995) binding to the same regulatory domain with different affinities. The most commonly used diterpene with phorbol ester structure is PMA. This phorboid has been extensively tested as a potent activator of PKC (Deleers and Malaisse, 1982). It binds to the C1B domain in PKC and promotes its translocation to the plasma membrane (Newton, 1995); unfortunately, it lacks of clinical use due to being a very potent tumor promoter (Szallasi and Blumberg, 1991). Its tumor-promoting activity seems to be associated to the nature and extent of the reversibility of PKC activation (Newton, 2018b), which in parallel is associated to the affinity of this molecules for the C1B domain and the effect of these molecules on the translocation of PKC to the plasma membrane. Thus, phorbol esters with more lipophilic substituents have a higher affinity for the C1B domain and lock PKC in an open (active) conformation on the membrane. This results in their dephosphorylation and subsequent degradation of the protein, a process referred to as down–regulation (Hansra et al., 1999). However, other diterpenes with a more hydrophobic character (Wang et al., 1999; Wang et al., 2000; Braun et al., 2005) reversibly activate PKC and this translates into signaling events that generate different kinds of cell responses (Murphy et al., 1999). This is the case of prostratin (13-*O*-acetyl-12-deoxyphorbol) a commercially available 12-deoxyphorbol initially isolated from the plant *Homalanthus nutans* (Márquez et al., 2008) or other 12-deoxyphorbols isolated from plants of the *Euphorbia* genus like ER272 (12-deoxyphorbol-13-isobutyrate) (Schmidt and Evans, 1977; Fatope et al., 1996; Kirby et al., 2010). The mechanisms underlying tumor promoting activity of PKC remain unknown, although it is possible that this downregulation is responsible for the tumor promoting activity (Newton, 2018b). Therefore, as it has been previously proposed by Newton (2018a,b), phorbol esters with more hydrophobic side chains like prostratin or other 12-deoxyphorbols (Schmidt and Evans, 1977; Fatope et al., 1996; Kirby et al., 2010) may lead to balance PKC activity generating the desired biological responses in the absence of tumorigenic activity.

Protein kinase C inhibitors have also been used in the literature to treat specific CNS injuries (Wang et al., 2014; García-Bernal et al., 2018; Tang et al., 2018). Specific inhibitors that target one PKC isozyme can only be found for classical PKCß (Lesyk et al., 2015) and atypical PKCζ (Puls et al., 1997; Bogard and Tavalin, 2015). On the contrary the majority of commercially available PKC inhibitors target a group of isozymes showing a smaller IC50 for classical PKCα and β. No specific inhibitors target novel PKCs alone without affecting other classical isozymes. Classical PKC inhibitors have been used to repair cervical dorsal spinal hemisection (Wang et al., 2014) and mechanical cortical injuries (García-Bernal et al., 2018), whereas peptide induce inhibition of novel PKCδ has been effective in the treatment of damage of the blood brain barrier (Tang et al., 2018). The molecular mechanisms underlying the effects of the inhibitors are not clear in some cases, because of the lack of specificity.

## Diterpenes, PKC and Neurogenesis

Non-tumor promoting diterpenes with 12-deoxyphorbol or lathyrane structure exert a proliferative effect on NPC cultures *in vitro* (Geribaldi-Doldan et al., 2016; Murillo-Carretero et al., 2017). Interestingly, whereas 12-deoxyphorbols promote NPC proliferation *in vitro* independently of their structure, not all lathyranes are able to exert this effect and only 3,12-di-O-acetyl-8-O-tigloilingol (ELAC) induces a classical PKC dependent effect on NPC proliferation. Treatment of NPC cultures with 12-deoxyphorbols in the absence of EGF increases proliferation promoting cyclin D and E expression mimicking the effects of EGF signaling. This sustains the hypothesis of a PKC dependent release of EGFR ligands (Geribaldi-Doldan et al., 2016; Murillo-Carretero et al., 2017). Additionally, intracerebroventricular administration of diterpenes such as the 12-deoxyphorbols like prostratin or ER272, and the diterpene with lathyrane skeleton ELAC, increase NPC proliferation in neurogenic niches in the adult mouse brain in a PKC dependent manner (Geribaldi-Doldan et al., 2016; Murillo-Carretero et al., 2017). The cellular and molecular mechanisms underlying the proliferative effect of these compounds have also been investigated and it is the specific activation of classical PKCβ what promotes the proliferation of EGFR^+^ transit amplifying cells in the SVZ (Murillo-Carretero et al., 2017).

## Conclusion

Regulating EGFR activity might be relevant when developing strategies to promote endogenous neurogenesis in brain injuries. A limiting step in this activation is the metalloprotease ADAM17, which is regulated by PKC. We have discussed in here how different PKC isozymes govern different steps of the neurogenic process in different niches, concluding that PKC might be a target to promote neurogenesis in injuries. Non-tumorigenic diterpenes with 12-deoxyphorbol or lathyrane skeleton activate PKC and increase NPC proliferation in adult neurogenic niches by activating classical PKC. Diterpenes with the capacity to activate classical PKC might be the active principle of useful drugs to treat disorders that require potentiation of neurogenesis (aging, AD, or Huntington’s disease among other). Because of their capacity to reversibly activate PKC, diterpenes are better drugs than other PKC activators that promote tumor growth. However, specific activators of each PKC isozymes need to be discovered in the short future that could be used to treat diverse CNS disorders in which PKC isozymes are differentially expressed and regulated. On the contrary, inhibition of classical PKC in injuries, thus maintaining novel PKC activities may lead to a limited release of EGFR ligands favoring the generation of new neurons over glial cells. Therefore, finding non-tumorigenic activators of each specific PKC isozyme, which facilitate adjustment of the homeostatic balances of PKC activity, will definitely lead to the development of new drugs and therapeutic strategies to regenerate brain injuries.

## Author Contributions

CC, NG-D, and PN-A contributed to conception and design of the review. NG-D wrote the first draft of the manuscript and created the cartoon in Figure 1. CC, NG-D, PN-A, RG-O, and SD-G wrote sections of the manuscript. RG-O organized all the information included in the table. All authors contributed to manuscript revision, read and approved the submitted version.

## Conflict of Interest Statement

The authors declare that the research was conducted in the absence of any commercial or financial relationships that could be construed as a potential conflict of interest.
